# Computational Cardiology — A New Discipline of Translational Research

**DOI:** 10.1016/j.gpb.2016.08.001

**Published:** 2016-08-03

**Authors:** Benjamin Meder, Hugo A. Katus, Andreas Keller

**Affiliations:** 1 Institute for Cardiomyopathies, Department of Internal Medicine III, University of Heidelberg, 69120 Heidelberg, Germany; 2 German Centre for Cardiovascular Research (DZHK), Heidelberg/Mannheim, Germany; 3 Chair for Clinical Bioinformatics, Medical Faculty, Saarland University, 66123 Saarbrücken, Germany

Over the past two decades, improved diagnosis, pharmaceutical therapies, and interventional strategies have impressively improved the armamentarium of modern cardiologists in the fight against the most incident and lethal diseases: heart failure, ischemic heart disease, and arrhythmia. The innovations in the field have mostly been enabled by inventions based on hypothesis-driven approaches. The invention and development of key cardiac biomarkers, such as natriuretic peptides and cardiac-specific troponins, may serve as examples. Based on few candidate molecules, the discovery of these markers requires neither high-throughput molecular screening, nor advanced computational methodologies for interpretation and refinement of results.

What has changed such that authors of this Special Issue on Computational Cardiology propose the requirement of a structured interaction of clinical, molecular, and bioinformatics experts? Evidence-based changes that have been implemented into current guidelines of cardiovascular medicine are mostly due to large-scale randomized clinical trials. Although this approach was hugely successful to improve prognosis of patients, in the future the treatment and outcome net-benefit expected from additional or refined treatments are predicted to become smaller and smaller. To further improve the management of heart disease, it is therefore important to increase personalization of new approaches. While the intricate power of statistics in randomized trials is obvious, the less than handful selection and phenotyping criteria (*e.g.*, age, body mass index, and kidney function) in such trials rather neglect the individuality of patients and their diseases. Hence, it is pivotal to enhance the characterization of prevalent and incident cardiovascular diseases, and either better select the appropriate therapy for the individual patient or define the personal drug target in a single patient.

One could list the selected foreseen challenges and roles of computational cardiology including:

• the identification of novel biomarkers with unambiguous information on diseases;
• the prediction of outcome (intraprocedural and postprocedural) for existing and upcoming pharmacotherapies and cardiac interventions;
• a real-time approach for data integration in the hospital setting and usage of big data on a population level;
• prevention of disease by early identification of health hazards;
• endorsement of health economic approaches in high-burden diseases, such as heart failure;
• improvement of point of care phenotyping and decentralized patient care to relieve the hospital setting;
• definition of new or personal drug-targets, *e.g.*, that are suited for gene repair;
• automated monitoring of novel treatments for the early detection of treatment success and side effects.

Based on these fields of future innovations, the vision of the future of precision medicine demands for far more than rocket science, namely the simulation of molecular pathways, cells, tissues, organs, and whole organisms. The ignition will be set by advances in molecular and clinical phenotyping in conjunction with the recent developments in bioinformatics methods for integration of multi-level high-throughput and high-content datasets with clinical data. This will pave the way for integrative approaches for patient care in the real sense of personalized medicine.

In this Special Issue on Computational Cardiology, we cover some of the exciting insights into new approaches toward a more personalized patient care using computational means. The authors highlight the importance of biobanks and comprehensive data resources, building the basis for future developments of diagnostics and potentially more targeted therapies [1], [2] and discuss the impact of quality control in next-generation sequencing data [3]. Beyond DNA-based approaches, they underline the relevance of transcriptomics, including gene expression, circular RNAs, and microRNAs [4], [5], [6]. In a Research Highlight, the potential of protein-coding sequences in the non-coding transcriptome is discussed [7]. Finally, more complex data integration approaches are described [8], leading to the whole-heart simulation of diastolic dysfunction and heart failure [9].

## Competing interests

The authors have declared no competing interests.
